# Cordycepin for Health and Wellbeing: A Potent Bioactive Metabolite of an Entomopathogenic Medicinal Fungus *Cordyceps* with Its Nutraceutical and Therapeutic Potential

**DOI:** 10.3390/molecules25122735

**Published:** 2020-06-12

**Authors:** Syed Amir Ashraf, Abd Elmoneim O. Elkhalifa, Arif Jamal Siddiqui, Mitesh Patel, Amir Mahgoub Awadelkareem, Mejdi Snoussi, Mohammad Saquib Ashraf, Mohd Adnan, Sibte Hadi

**Affiliations:** 1 Department of Clinical Nutrition, College of Applied Medical Sciences, University of Hail, Hail PO Box 2440, Saudi Arabia; amirashrafy2007@gmail.com (S.A.A.); ao.abdalla@uoh.edu.sa (A.E.O.E.); mahgoubamir22@gmail.com (A.M.A.); 2 Department of Biology, College of Science, University of Hail, Hail PO Box 2440, Saudi Arabia; arifjamal13@gmail.com (A.J.S.); snmejdi@yahoo.fr (M.S.); 3 Bapalal Vaidya Botanical Research Centre, Department of Biosciences, Veer Narmad South Gujarat University, Surat 395007, Gujarat, India; patelmeet15@gmail.com; 4 Laboratory of Bioresources: Integrative Biology and Valorization, (LR14-ES06), University of Monastir, Higher Institute of Biotechnology of Monastir, Avenue Tahar Haddad, BP 74, Monastir 5000, Tunisia; 5 Department of Clinical Laboratory Sciences, College of Applied Medical Science, Shaqra University, Al Dawadimi PO Box 17431, Saudi Arabia; ashrafsaquib@gmail.com; 6 School of Forensic and Applied Sciences, University of Central Lancashire, Preston PR1 2HE, UK

**Keywords:** *Cordyceps*, cordycepin, medicinal mushroom, nutraceutical, anti-diabetic, immunomodulator, anti-hyperlipidemia, Chinese medicine, DongChongXiaCao, bioactive compound

## Abstract

*Cordyceps* is a rare naturally occurring entomopathogenic fungus usually found at high altitudes on the Himalayan plateau and a well-known medicinal mushroom in traditional Chinese medicine. *Cordyceps* contains various bioactive components, out of which, cordycepin is considered most vital, due to its utmost therapeutic as well as nutraceutical potential. Moreover, the structure similarity of cordycepin with adenosine makes it an important bioactive component, with difference of only hydroxyl group, lacking in the 3′ position of its ribose moiety. Cordycepin is known for various nutraceutical and therapeutic potential, such as anti-diabetic, anti-hyperlipidemia, anti-fungal, anti-inflammatory, immunomodulatory, antioxidant, anti-aging, anticancer, antiviral, hepato-protective, hypo-sexuality, cardiovascular diseases, antimalarial, anti-osteoporotic, anti-arthritic, cosmeceutical etc. which makes it a most valuable medicinal mushroom for helping in maintaining good health. In this review, effort has been made to bring altogether the possible wide range of cordycepin’s nutraceutical potential along with its pharmacological actions and possible mechanism. Additionally, it also summarizes the details of cordycepin based nutraceuticals predominantly available in the market with expected global value. Moreover, this review will attract the attention of food scientists, nutritionists, pharmaceutical and food industries to improve the use of bioactive molecule cordycepin for nutraceutical purposes with commercialization to aid and promote healthy lifestyle, wellness and wellbeing.

## 1. Introduction

*Cordyceps*, derived from two Latin words “cord” and “ceps” representing ‘club’ and ‘head’ respectively, describing it as club fungi. It is an entomopathogenic fungus where extensions of the stroma and fruiting body arise from insect larvae carcasses [1]. *Cordyceps* predominantly lives on the head of larvae of a particular moth species, *Hepialus armoricanus Oberthur* (Lepidoptera). It belongs to the Ascomycetes family and has been a very well-known fungus in Chinese traditional medicine for the last 300 years. *Cordyceps* is also known as ‘Dong Chong Xia Cao’, which means ‘Worm in winter and grass in summer’ in China [2,3,4]. According to the previous reports, around 1200 types of entomopathogenic fungi are known, out of which, *Cordyceps* is considered as one of the largest genus containing approximately 500 species. Several species of *Cordyceps* have been cultivated for their therapeutic properties such as *Cordyceps sinensis, Cordyceps sobolifera, Cordyceps cicadicola, Cordyceps liangshanesis, Cordyceps ophioglossoides and Cordyceps militaris* [5]. On the other hand, keeping in mind the therapeutic value, its major distribution location at approximately 14,000 ft altitude in the Himalayan regions of China, Nepal, Tibet and India makes it very expensive at around USD ($) 12,000 kg^−1^ [3,6,7]. Moreover, despite the harvesting difficulties and distribution, it is still considered a highly valued mushroom because of its abundant natural bioactive component resources with various potent biological activities and nutraceutical importance [2]. For hundreds of years, *Cordyceps* were used as a folk tonic food, but only in recent times, its potential pharmaceutical as well as nutraceutical application have been explored, which has attracted food scientists globally [8].

Currently, it has been observed that a majority of the population from developed as well as developing countries are suffering from chronic diseases, and the underlying causes are believed to be rapid urbanization and changes in eating and lifestyle behavior. Among the various underline causes, eating habits are considered one of the major risk factors for chronic diseases, such as obesity, diabetes, hypertension, hyperlipidemia and many more affecting both wellness and the wellbeing of mankind [9,10]. Therefore, the scientific community is working relentlessly to develop naturally occurring or naturally derived product, such as nutraceuticals, which could help in improving the human health status while not possessing harmful effects. *Cordyceps* are among the thousands of mushroom available containing various bioactive components with innumerable health benefits [11]. It has been used for its therapeutic values since long time and new promising features of cordycepin-based nutraceuticals are an advantage for the current population. There is a very well-known quote from Hippocrates stating that, “Let food be thy medicine and medicine be thy food”, describing the importance of nutrition for the prevention, treatment and management of diseases. Therefore, *Cordyceps,* as an edible mushroom, could be an ideal nutraceutical containing both nutritionally bioactive components as well as a source of various physiological benefits [12,13]. Moreover, based on our literature search, we found that researchers have majorly discussed cordycepin for its anticancer potential, but other therapeutic applications and potential nutraceutical approaches have either not been discussed in detail or ignored. The main objective of this review is to focus on the nutraceutical potential of cordycepin (the major bioactive component of *Cordyceps*), using a mechanistic approach to study its pharmacological functions as well as to demonstrate the benefit of commercial availability of cordycepin-based nutraceuticals.

## 2. Nutritional Value of *Cordyceps*

Nutrition is considered as a fundamental pillar of human beings for maintaining health or in development across the entire life span. Moreover, it is very important to have a proper diet and enough nutrition for survival, physical growth, mental development, performance and productivity, health and well-being [14]. One often quoted phrase, “medicines and foods have a common origin” and based upon this idea, *Cordyceps* could be considered as one of the most significant mushrooms, enriched with various nutrients with possible nutraceutical value [12]. Abundant amounts of bioactive components are present in *Cordyceps* such as proteins, fats, essential amino acids, volatile oils, carotenoids, phenolic compounds, flavonoids, minerals (Fe, Ca, Mg, Ni, Sr, Na, Ti, Pi, Se, Mn, Zn, Al, Si, K, Cr, Ga, V and Zr), vitamins (B1, B2, B12, E and K) as well as various types carbohydrates like monosaccharides, oligosaccharides, polysaccharides, sterols, nucleosides, etc. [15,16,17,18,19]. Proximate analysis of some of the *Cordyceps* species have reported that moisture, total ash, crude protein, fat, crude fibre and carbohydrate content are 7.18%, 7.48%, 21.46%, 1.80%, 6.40% and 55.68%, respectively [20]. Many authors have also reported proximate analyses of *Cordyceps* fruiting bodies and mycelial biomass. The protein, moisture, ash, fat, and carbohydrate compositions of *Cordyceps* fruiting bodies were reported as 59.8%, 5.7%, 5.1%, 8.8% and 29.1%, whereas, mycelial biomass contains 39.5%, 13.1%, 5.7%, 2.2% and 39.6% of protein, moisture, ash, fat and carbohydrates, respectively [21]. On the other hand, the amino acid contents of the corpus as well as fruiting bodies of *Cordyceps militaris (C. militaris)* were reported to be 14.03 mg/g and 69.32, respectively. Additional amino acid analysis indicates that the fruiting bodies contains abundant amounts of proline, lysine, threonine and glutamic acid. Moreover, fatty acid profiling indicates almost 70% of unsaturated fatty acids out of the total fat percentage. Importantly, the amount of cordycepin and adenosine in both corpus and fruit bodies was reported to be (0.97 and 0.36%) and (0.18 and 0.06%), respectively [17,22]. With the increasing interest in *Cordyceps* in recent times for pharmaceutical, nutraceutical or food biotechnological purposes, further research is necessary to obtain an overview of the potential of this medicinal mushroom [2].

## 3. Bioactive Components of *Cordyceps*

**Figure 1:**
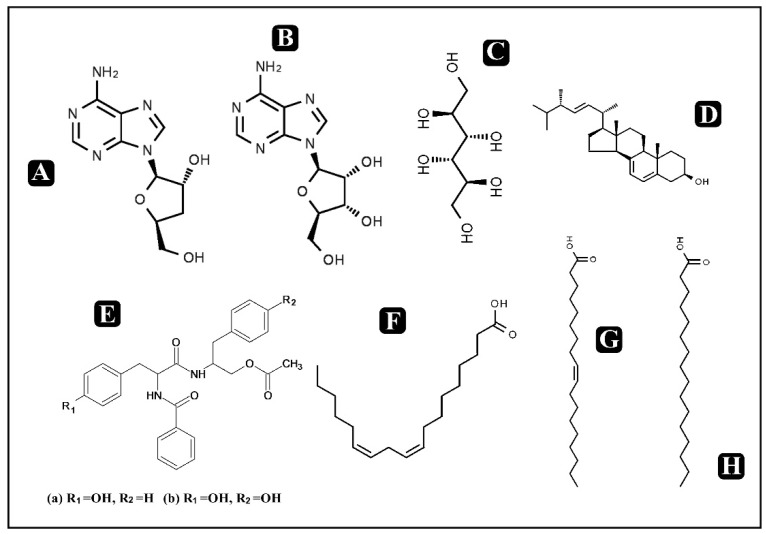
Chemical structures of some known and potent bioactive compounds in Cordyceps. (**A**) Cordycepin (**B**) Adenosine (**C**) Cordycepic acid (**D**) Ergosterol (**E**) Structure of (a) and (b). (a) Cordyceamides A (b) Cordyceamides B (**F**) Linoleic acid (**G**) Oleic acid (**H**) Palmitic acid.

**Figure 2:**
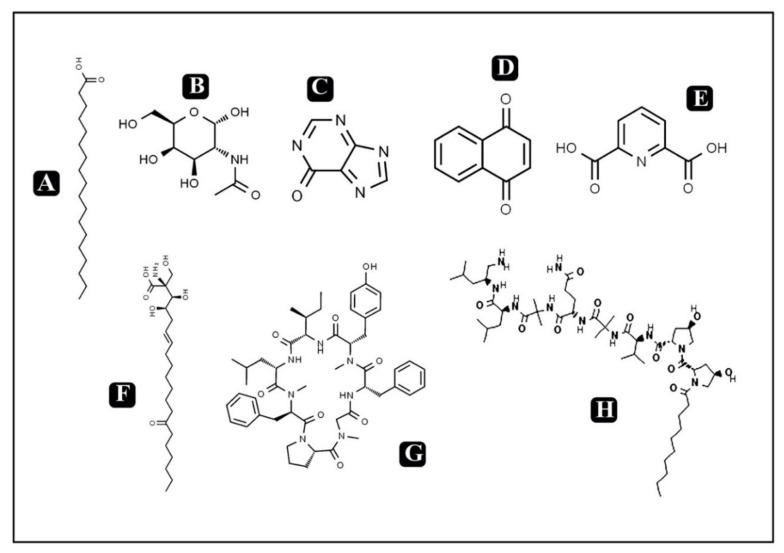
Chemical structures of some known and potent bioactive compounds in *Cordyceps.* (**A**) Stearic acid (**B**) *N*-acetyl muramic acid (**C**) Hypoxanthine (**D**) Nephthaquinone (**E**) Dipliconic acid (**F**) Myriocin (**G**) Cordyheptapeptide A (**H**) Cicadapeptin 1.

The significance of bioactive components present in foods has been gaining interest recently due to public health concerns. Moreover, with rising consumer awareness regarding promotion, prevention as well as maintenance of health, bioactive components present in food could play a very important role [23,24]. Bioactive compounds can be described as molecules present in food derived of plant or animal sources consumed to have a regular energy intake along with various therapeutic activities such as against metabolic disorders, anti-inflammatory, chronic diseases and many more [25]. The presence of these bioactive components, even in minute quantities, not only meets the basic nutritional needs, but also confers numerous health benefits. Epidemiological studies indicate that high consumption of foods rich in bioactive compounds with different phytochemicals such as antioxidants, vitamins, flavonoids and carotenoids has a positive effect on human health [26]. Mushrooms have been known and used for centuries for food and medicinal use. Among various mushrooms, very well-known medicinal mushrooms like *Cordyceps* producing bioactive metabolites are used or studied for the possible treatment of several diseases [15]. The presence of antioxidants acquired by modifying our diet with mushrooms could play an important role in the prevention of diseases. *Cordyceps* have a history of medicinal use spanning millennia in parts of Asia, but they can also be potentially used for their nutraceutical values [22,27]. *Cordyceps* have been reported to contain various bioactive components such as proteins, fat, carbohydrates, exopolysaccharides, cordycepin, phenolic compounds, polysaccharides, cordycepic acid, adenosine, proteoglucans, terpenoids, amphinol, steroids, ergosterol, lectins, etc. A detailed list of bioactive components present in *Cordyceps* is presented in Table 1 along with the chemical structures of some potent identified bioactive molecules (Figure 1 and Figure 2). Out of these, cordycepin is the main active constituent which is most widely studied for its medicinal value along with its nutraceutical potential [2,8,22,28,29]. Moreover, various mechanisms have been reported for the pharmacological actions of cordycepin such as inhibition of DNA and RNA synthesis, post-transcriptional processing of hnRNA and activation of adenylate cyclase, inhibition of chemotaxis and particular protein synthesis of macrophage cell lines, anti-tumorigenic activity on some cell lines, enhancement of cell differentiation etc. [1].

## 4. Cordycepin and Its Chemical Features

Cordycepin (C_10_H_13_N_5_O_3_, molecular weight 251.24 Da, melting point 228–231 °C) is chemically (9-(3-deoxy-β-D-ribofuranosyl) adenine), 3’–deoxyadenosine [5,47]. The structure of cordycepin consists of a purine molecule attached to one ribose sugar moiety [48]. The NMR spectrum of cordycepin shows a singlet at 3.4 ppm, attributed to the C-H proton and a -NH_2_ peak reported to be present at 4.6 ppm, whereas, the peaks due to the different -OH groups are found to in the 8–8.5 ppm range [47]. The concentration of cordycepin in *C. militaris*, i.e., around 2–3 mg/kg, is very low compared to the needs of the commercial market, therefore various synthetic and semi-synthetic methods have been reported for the preparation of cordycepin [49]. Cordycepin can be synthesized chemically by replacing an OH group with an H group at the 3′-position of the ribofuranosyl moiety to produce an adenosine analogue [50]. Cordycepin is found to be structurally very similar to adenosine except for the lack of a 3′-hydroxyl group [49,51] Due to its very close structural similarity with adenosine, it is considered to be a very potent bioactive component with essential properties for its nutraceutical applications. There are various reports suggesting that cordycepin competitively inhibits the courses of synthesis and metabolism of DNA and RNA, as well as it affects the activity of adenosine deaminase and the mTOR signaling pathway [52]. Therefore, cordycepin has been reported to have a wide variety of pharmacological actions such as antioxidant, immunological, hypolipidemic, anti-inflammatory, anticancer, antimicrobial, antiviral, and hypoglycemic properties [49].

## 5. Cordycepin as a Nutraceutical and Its Role in Human Health

**Figure 3:**
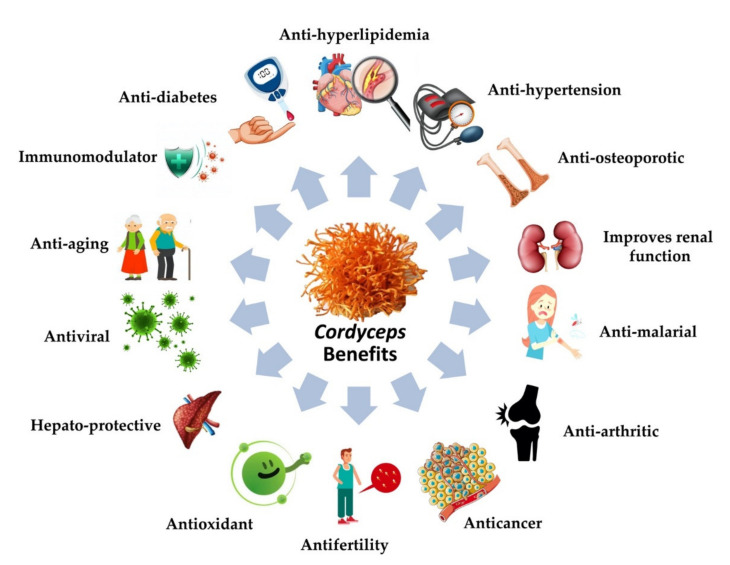
Pictorial representation of *Cordyceps* therapeutic potential in general.

The concept of “nutraceutical” was first introduced in the survey study conducted in France, United Kingdom and Germany, where they found that rating of diet by the consumers was much higher compared to the other factors such as exercise or hereditary to achieve a good health [53]. DeFelice was the first to introduce the word “nutraceutical” by coalescing the two words “nutrition” and “pharmaceutical” and define it as “food or a part of food which not only impart health benefits but also contributes in preventing or treating various diseases”. Moreover, in broad terms nutraceuticals can be summarized as bioactive components which play a vital role in human beings by maintaining their normal physiological functions and well-being [10,54]. Notably, nutraceuticals have been designed in such a way, that it could be useful for the betterment and maintenance of human health without causing any harm due to their natural occurrence [55,56]. Nutraceuticals of plant, animal origin or live microorganisms offer great opportunities for food scientists and food industries to produce novel foods or food components for the future needs of human beings to stay healthy [57,58]. Nutraceuticals have been classified in various ways based upon their understanding and applications. Naturally available food sources are considered for their nutraceutical values. They can be characterized as antioxidants, dietary fibre, prebiotics, polyunsaturated fatty acids, probiotics, vitamins, polyphenols and spices [9,53]. The sudden rise in demand of nutraceutical products is mainly due to their therapeutic properties in various ailments such as the common cold, hypertension, heart disease, arthritis, dyslipidemia, diabetes, cancer, depression, inflammatory bowel disease, and increased life span by postponing aging, integrity of the body and support of smooth normal functioning. Therefore, nutraceuticals could play an important role in the improvement of health as well in chronic disease prevention [58]. Nutritionally potent *Cordyceps* are considered a powerhouse of energy, because of their ability to revitalize several organ systems. Moreover, according to various scientific reports, the *Cordyceps* active component cordycepin is considered very useful due to its potential application in various ailments (Figure 3). Several pharmaceutical as well as nutraceutical preparations made from *Cordyceps* dry powder (Table 2) are marketed and reported to protect renal and hepatic functions, improve intracellular energy exchange, increases oxygenation and natural endurance, remove toxins from the body, control blood glucose level and lipid profile, delays the aging process, stimulates the natural metabolism of energy and nourishes the body’s immune system [59].

## 6. Mechanism of Action and Pharmacological Implications of Cordycepin

### 6.1. Cordycepin and Diabetes

Diabetes has recently become one of the most prevalent epidemics worldwide, affecting almost 382 million people. According to the reports, it is believed that every year approximately 1.3 million people die from diabetes. According to International Diabetes Federation (IDF), it is estimated that around 629 million people will be diabetic around the globe by 2045. However, as per the recommendation from dieticians or physicians, a healthy life style and healthy food habits, could be one of the key answers to this problem [60,61]. It has also been observed that, in most diabetic patients, several other complications also arise, such as cardiovascular diseases, retinopathy, nephropathy, hyperlipidemia and neuropathy [62]. Of note, it is also suggested by various studies, that no single treatment programme can treat diabetes, although most of the treatments achieve normal blood glucose levels or improve microcirculation [63]. Currently, pharmaceutical products used for the management or therapeutic purposes against diabetes are sulfonylureas, biguanides, thiazolidinedione, α-glucosidase inhibitors or insulin injections. However, pharmaceutical products available for the treatment of diabetes have several adverse effects and their potency is sometimes controversial. Other non-medicinal strategies used in diabetes are exercise, weight loss plans and changes in food habits. Occasionally, complications of diabetes can cause morbidity because of pathophysiology flaws. Therefore, consumer interest has currently moved towards alternative medicinal approaches such as nutraceutical food products containing bioactive antidiabetic components. Some studies have reported that extract of *C. militaris* showed a significant decrease in blood glucose levels by virtue of increasing glucose metabolism as well as protection against diabetic nephropathy [64].

**Figure 4:**
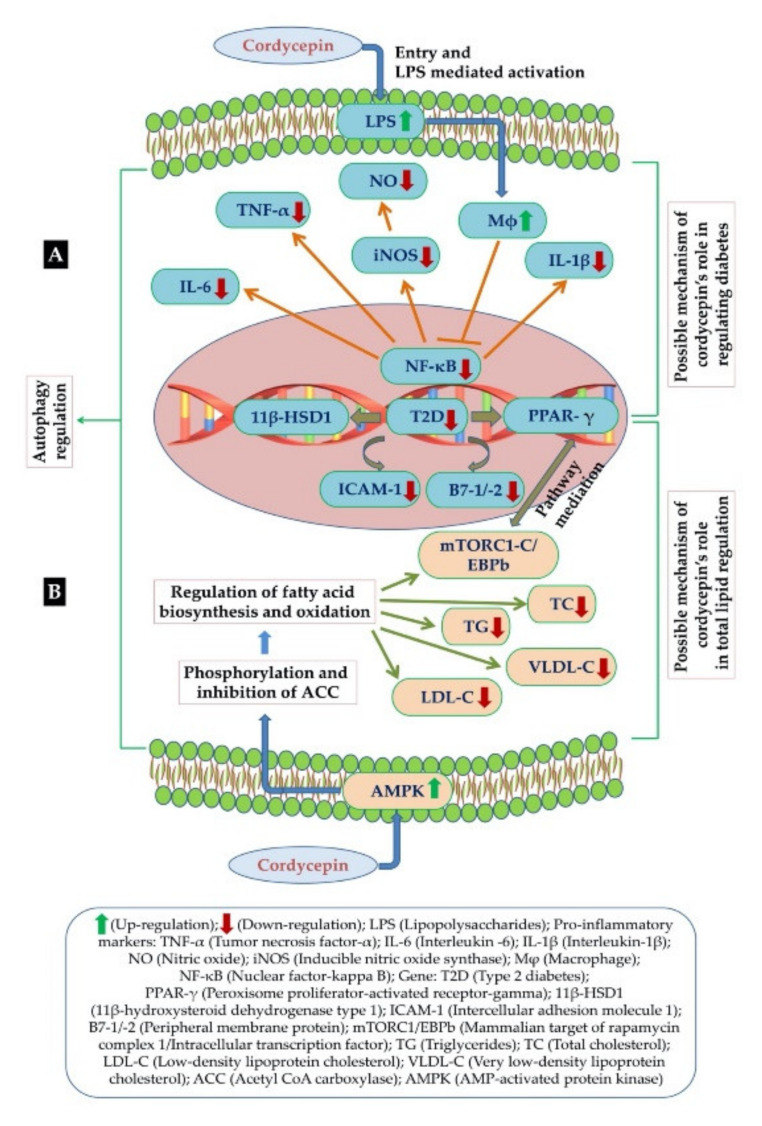
(**A**) Possible mechanism of cordycepin for its anti-diabetic activity (**B**) Possible mechanism of cordycepin in regulation of fat metabolism in hyperlipidemia [47].

The mechanism of cordycepin’s antidiabetic activity is not fully understood, but a few studies have explained a possible pathway. They found that cordycepin prevents the production of NO and pro-inflammatory cytokines like TNF-α, IL-1β, and IL-6 in LPS activated macrophages by inhibiting the protein expression of pro-inflammatory mediators. By virtue of this the expression of type 2 diabetes-regulating genes (11β-HSD1 and PPARλ) was reduced. Expression of co-stimulatory molecules such as ICAM-1 and B7-1/-2 was also decreased with the increase in cordycepin concentration as presented in Figure 4A [65]. Furthermore, cordycepin has been found to suppress the expression of diabetes-regulating genes through the inactivation of NF-κb-dependent inflammatory responses [66,67]. In another study, cordycepin’s antidiabetic activity was reported in an alloxan-induced diabetic mouse model. The results suggested a significant improvement in glucose tolerance tests after administration of an effective dose of cordycepin [68]. Additionally, an effect of cordycepin on diabetic nephropathy by suppressing cell apoptosis, renal fibrosis and rescued cell autophagy in the diabetic nephropathy rat model was also reported [69]. There are several reports which suggest that cordycepin has very good potential for being a safe anti-diabetic pharmaceutical agent [64,65,70].

### 6.2. Cordycepin and Cardiovascular Diseases (Hyperlipidemia)

Cardiovascular diseases have become one of the major causes of death around the world in developed as well as developing countries, and it has been assumed that there are various factors (excessive intake of tobacco, alcoholism, sedentary lifestyle, unhealthy eating habits etc.) associated with it. Among the various risk factors, rises in lipid levels are believed to be one of the main ones causing this chronic disease [71]. Hyperlipidemia is caused by or considered as the amount of fatty acids present in lipids, low density lipoprotein cholesterol, trans fats and triglycerides accumulated in human bodies causing cardiovascular diseases such as atherosclerosis and coronary heart disease [72,73,74]. Moreover, a mechanistic approach involved in the regulation of fat metabolism is due to AMP-activated protein kinase (AMPK), a main cell electricity sensor [75]. In addition, activation of AMPK causes a decline in levels of fatty acids through phosphorylation and inhibition of acetyl-CoA carboxylase (ACC), which helps in regulation of fatty acid biosynthesis and oxidation (Figure 4B). AMPK activation was also reported to decrease total cholesterol and triglycerides by inhibiting the activity of glycerol-3-phosphate acyltransferase (GPAT) and HMG CoA reductase, the two rate-limiting enzymes in TC and TG synthesis, respectively [76]. Wu et al. [77] proposed that cordycepin could additionally prevent intracellular lipid accumulation through activation of AMPK interplay with the Ɣ1 subunit [77]. Thus, it was found that regulation of AMPK would provide a solution for overweight and obese people causing hyperlipidemia [78]. According to previous reports, cordycepin has been found very effective in lipid reduction, due to its chemical structural similarity with adenosine (an activator of AMPK). Similarly, administration of cordycepin was able to reduce the accumulation of low-density lipoprotein cholesterol, total cholesterol and triglycerides effectively, and could be a potent nutraceutical agent for reducing hyperlipidemia caused by high fat diets [79,80]. On the other hand, cordycepin was also evaluated for regulating autophagy as well as lipid metabolism. It has been found that cordycepin was effective against hepatic lipid accumulation induced by PA through autophagy induction and PKA/mTOR pathway could be the possible mechanism behind its efficacy. More importantly, it was observed that cordycepin was largely effective in reducing the intracellular levels of total lipids, total cholesterol, C and TG, LDL-C, VLDL-C as well as LDL-C/HDL-C and TC/HDL-C ratios. Therefore, cordycepin lipid-lowering activity could be of potential use in the treatment of hyperlipidemia [77,79,81].

### 6.3. Cordycepin and Anti-Inflammatory Effects

Inflammation, a natural response to injury, occurs in our body to eliminate harmful elements such as damaged cells, irritants and pathogens by initiating the healing system. Acute and chronic pulmonary inflammations have been reported in various respiratory diseases such as asthma, acute respiratory distress syndrome, cystic fibrosis (CF) and chronic obstructive pulmonary disease (COPD) [82]. Of note, cordycepin has been reported to suppress intestinal irritation in an acute colitis mouse model as well as in microglia through inhibiting pro-inflammatory mediators such as TNFα [83,84]. It has been found that cordycepin is effective in a mouse version of bronchial asthma as well as it improves both mucus clearance and airway surface hydration, as it is hyper-secreted in various respiratory problems, like COPD and asthma [85,86]. In addition, cordycepin has also been reported to attenuate airway remodeling in rats with COPD by preventing airway inflammation as well as TGF-β1/Smad signaling pathway. According to this report, cordycepin could be useful in the case of COPD [87,88]. Cordycepin extracts were reported to relieve fibrosis in the lung by inhibiting TGF-b1 expression [89,90] and the promotion of collagen degradation [91]. Therefore, based upon these collective reports and data, it could be summarized that cordycepin has all the potential to become a very potent bioactive anti-inflammatory component [83,84,85,92].

### 6.4. Cordycepin and Immunomodulatory Effects

Immunomodulation is usually defined as modulation of the immune system, and this can be done by any chemical agent that modifies the immune response or the functioning of the immune system via the stimulation of antibody formation or the inhibition of white blood cell activity [93]. Cordycepin has been reported to stimulate cytokine release of resting peripheral blood mononuclear cells (PBMCs) as well as influence PBMCs proliferation and transcription factors in a human acute monocytic leukemia cell line (THP-1). Moreover, cordycepin was found to regulate human immune cells functions in vitro [94,95]. It has also been observed that the antitumor activity of cordycepin is associated with its immunomodulatory effects [96]. Additionally, pure bioactive components extracted from *C. militaris* are reported to have good immunomodulatory effects by increasing the survival rate of lupus mice and reducing anti-ds-DNA production [34,65]. Zhang and Xia [97] observed that *C. sinensis* behaves like an immunosuppressant in a heterotopic heart allograft model in rats and increases the survival period. The same effect has been reported by other authors too, for example, Zhu and Hu [98], revealed that *C. sinensis* prolongs the mouse skin allograft survival time. Therefore, cordycepin has been proven a potentially effective immunomodulator, and it is even specifically used to control autoimmune disorders and transplant rejections after an organ transplant [99]. An increasing number of studies indicate that cordycepin is a bi-directional modulator with both suppressive as well as influencing effects on the immune system by regulating the adaptive and innate immunity [1,100,101,102,103].

### 6.5. Cordycepin and Anti-Osteoporosis Effect

Osteoporosis is a condition of low bone mineral density (BMD) and loss of the structural and bio-mechanical properties of bones. It increases the risk of fracture, as bones become more porous and fragile. Osteoporosis mainly occurs in aged people, specifically in post-menopausal women and patients who had long-term treatment of steroid therapy. Anti-osteoporotic effect of cordycepin was studied in ovariectomized osteopenic rats, it has been found that cordycepin was able to counteract the loss of bone in the experimental model. The mechanistic approach used in this study showed the decline in activity of tartrate-resistant acid phosphatase and alkaline phosphatase enzymes both *in vitro* and *in vivo*. Moreover, the results showed that oral intake of cordycepin could increase the level of osteocalcin (OC), a marker of bone formation, and decrease C-terminal cross-linked telopeptide of type I collagen (CTX) level, a marker of bone resorption, as well as restore oxidative stress levels in ovariectomized rats [104]. These results suggest that cordycepin can be a valuable bioactive compound for the treatment of osteoporosis and is able to prevent bone loss caused by estrogen deficiency. Cordycepin was also reported to inhibit RANKL-induced osteoclast differentiation (RANKL), receptor activator of nuclear factor kappa-Β ligand, and down-regulate the mRNA expressions of osteoclastogenesis-related genes such as, matrix metalloproteinase (MMP)-9, cathepsin K, tartrate-resistant alkaline phosphatase (TRAP) and nuclear factor of activated T-cells, cytoplasmic 1 [105].

### 6.6. Cordycepin and Anti-Arthritic Effect

Arthritis, an autoimmune disease affecting bone joints, is mainly characterized by joint stiffness as well as joint pain, among other symptoms such as swelling, warmth, redness, and reduction in joint motility. There is no known specific effective treatment for arthritis, although many drugs such as glucocorticosteroids, non-steroidal anti-inflammatory drugs, and other biological agents are used to improve the symptoms, such as pain, fatigue, and disability. Long-term usage of these drugs decreases their effectiveness and increases side effects. Recently, studies were conducted looking for effective anti-arthritic drugs with increased therapeutic effects and fewer side effects. Traditional herbal medicine, which is shown to be more effective, safer, and economical, has attracted more attention in the area of arthritis treatment. Moreover, cordycepin has been found to modulate glycosaminoglycan (GAG) release by suppressing stimulation of IL-1β. In addition, levels of proteases that have been reported in cartilage matrix degradation, such as MMP-13, cathepsin K, MMP-1, cathepsin S, ADAMTS-5 and ADAMTS-4, were decreased by cordycepin in a dose-dependent manner. Chondroprotective effect of cordycepin by preventing cartilage denegation as well as interfering inflammatory response in osteoarthritis pathogenesis has also been reported [106]. Cordycepin has been reported to reduce excessive inflammatory cell infiltration via down-regulation of macrophages, interferon gamma-induced protein 10 (IP-10) and Mig expressions through terminating protein coding gene (STAT1) phosphorylation [107]. There are some reports suggesting that inflammation of T-cell infiltration could be inhibited by using a cordycepin concentration of 10 mg/kg. According to that report, cordycepin can regulate the T-cell receptor, a protein complex found on the surface of T-cells, that signals to suppress excessive T-cell activation in inflammation [108]. Therefore, based upon these reports, it can be concluded that cordycepin has therapeutic potential in both anti-catabolic and anti-inflammatory actions against arthritic diseases [107,108].

### 6.7. Cordycepin and Antioxidant Effect

Antioxidants are compounds that can prevent or slow down oxidation reactions producing free radicals, that ultimately cause cell damage in organisms. Oxidative stress, which is related with an increased formation of oxidizing species or a significant reduction of natural antioxidant levels, is involved in different human diseases (cellular necrosis, cardiovascular disease, cancer, neurological disorder, ageing) [109]. Non-toxic antioxidants from natural sources, particularly medicinal plants, are known to prevent oxidative damage due to their richness in polyphenolics and bioactive compounds [110,111,112,113,114]. The antioxidant activity of *Cordyceps* has been reported by various authors [115,116]. Cordycepin has been reported to significantly increase the levels of antioxidant enzymes such as superoxide dismutase and glutathione peroxidase activities in 6-OHDA-treated cells. Moreover, the results showed that cordycepin prevents 6-OHDA-induced neurotoxicity in adrenal pheochromocytoma cells (PC12 cells) via its potent antioxidant action [117]. In addition, cordycepin containing protein-bound polysaccharide causes a reduction in lipid peroxidation as well as an increase in the activity of antioxidant enzymes in the liver like catalase and superoxide dismutase [118]. Other authors have suggested the potential of cordycepin in reducing lipid peroxidation in mouse liver [119]. Therefore, per these reports, cordycepin could be considered as a potential antioxidant. A few studies have even suggested that the antioxidant potential of *Cordyceps* is close to that of ascorbic acid [120].

### 6.8. Cordycepin and Anti-Malarial Effect

Malaria, a very common disease globally with a high mortality rate, is caused by *Plasmodium*, a parasitic organism [121,122,123,124]. The parasite gets into the human body through the bites of infected mosquitoes [125,126,127]. It is one of the deadliest diseases in the world [128], as it is estimated that every 2 min, a child dies of malaria, and each year more than two hundred million new cases of the disease are reported [129]. Most of the people who die from the disease are young children in Africa. The effects of cordycepin on the malaria parasite in mice were first studied by Trigo et al. [130]; they suggested that the growth of the parasite was affected by cordycepin that affects the nucleic acid and protein synthesis of the parasite. This makes cordycepin a possible molecule which can be explored further as a probable anti-malarial agent.

### 6.9. Cordycepin and Other Diseases

Hyperuricemia is a long-time purine metabolic disorder recognized as a result of excessive serum uric acid status in blood and associated with gout, renal sicknesses, hypertension, hyperlipidemia, and atherosclerosis [96,131]. *C. militaris* has been reported for its anti-hyperuricemic effect in hyperuricemic mice at different doses, reaching the levels of normal mice [132]. In another study, Yong et al. [133] also reported cordycepin’s potential as an anti-hyperuricemic in hyperuricemic mice. Infertility can be described as a disease condition where females are not able to become pregnant despite having frequent, unprotected sex for at least a year for most couples. According to the reports, cordycepin has been proved potent in increasing both the sperm quality and quantity. *C. militaris* supplementation has been stated to bring about an increase of serum cordycepin concentration, which concurrently enhances testosterone and estradiol-17 levels, ultimately increasing the percentage of motile sperm cells [134]. In addition to this cordycepin is also reported to increase semen production as well as sperm quality in boars [66]. The effect of cordycepin on testosterone levels in male rats was reported. It was found that the concentration of testosterone in the serum of the rats was significantly increased by *C. militaris*. Therefore, fruiting bodies of *C. militaris* grown on the drone bee medium could act as an integrative medicine for the treatment of reproductive problems caused by insufficient testosterone levels in human males [135]. On the other hand, chronic kidney disease (CKD) is a disease condition where the condition of the kidneys deteriorates steadily and has been related with both non-communicable diseases, e.g., diabetes and hypertension, and infectious diseases like hepatitis B, malaria, and HIV [136]. Clinical research exploring the possible cordycepin application has confirmed the beneficial effects in decreasing the progression of end-stage kidney disease in CKD patients [137]. Moreover, other pharmaceutical applications of cordycepin are also recommended, such as increasing creatinine clearance, serum albumin and hemoglobin, lowering serum creatinine levels as well as improving lipid metabolism [137,138,139,140].

## 7. Global Nutraceutical Market of Cordycepin: Current and Future Trend

**Figure 5:**
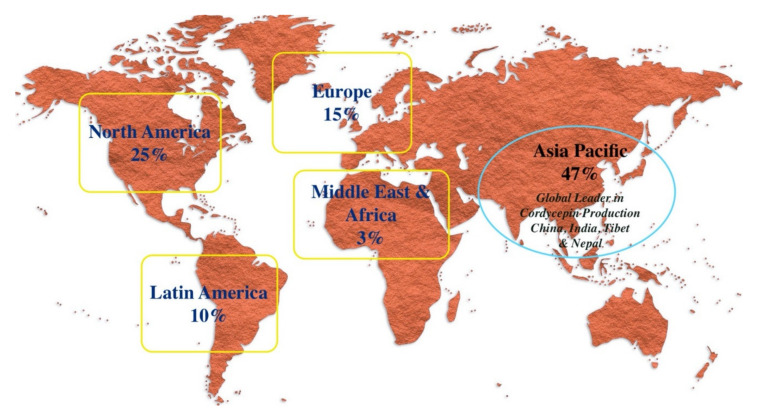
Expected global nutraceutical market by 2025 with China, India, Tibet and Nepal as global leaders for production and extraction of cordycepin [144].

Nutraceuticals are developed from foods or food derived components due to their health promoting properties to establish a clear link between ‘nutrients’ and ‘pharmaceuticals’ [9]. The health promoting effects of nutraceuticals have led to substantial increases in their market predominantly since the 1990s. Nutraceutical products have shown hope for various chronic diseases (diabetes, cancer, heart disease, hypertension, common cold, dyslipidemia, arthritis and many more) as well as help in delaying aging and eventually increasing life span [58]. Health awareness in consumers, caloric intake and weight management in countries like US, China and India, have already endorsed various applications of nutraceuticals, causing a significant impact on the industry growth. According to the data published online by the Grand View research team, the global nutraceutical market size could reach USD 722.49 billion by 2027, and is expected to expand at a CAGR of 8.3% over the forecast period [141]. Major key companies such as Abbott, Amway, Danone S.A., Nestle S.A., Glanbia Nutritionals, Herbalife International of America, Inc., The Archer Daniels Midland Company, BASF SE, PepsiCo, Inc., General Mills, Inc. are involved in the research & development of nutraceutical production [142]. On the other hand, the global cordycepin market is predicted to surpass US$ 1 billion by 2026. Asia Pacific (mainly China) is considered as the global leader in the production or extraction of cordycepin from *C. sinensis* and *C. militaris*, with an expected 47% of global nutraceutical market (Figure 5) [143].

## 8. Safety and Efficacy

*Cordyceps*, a well-known traditional Chinese medicine is also called a miracle mushroom because of its exceptional health benefits. Due to its natural occurrence, it is considered pharmacologically safe for human consumption. However, in some cases it has been reported to cause dry mouth, nausea, abdominal distension, throat discomfort, headache and diarrhea as well as allergic reactions [22,34,145]. It is recommended to avoid its consumption in the case of patients suffering from systemic lupus erythematosus, multiple sclerosis and rheumatoid arthritis. Moreover, there are a few reports indicating that *Cordyceps* intake can cause lead poisoning in some cases. Considering the potential for toxicity, resistance and its efficiency, potential drug derivatives designed from the natural products for the adenosine deaminase-resistant properties and low-toxicity causing effects of cordycepin, as well as organ-targeted nanoparticles for cordycepin delivery for *in vivo* therapy, are also needed [146]. Further clinical, experimental as well as epidemiological data are required for identification of other molecular targets to see the correlation between *Cordyceps* and other diseases like cancers and to estimate the validation of optimum dosing for its safety and efficacy [28]. The mechanism of action of cordycepin still needs further detailed scientific studies along with exploration of the various biochemical pathways involved. Thereby, its pharmacological and mechanism of actions will help in scientific scrutiny to answer every aspect of cordycepin [34]. Aside from some negative reports and clinical data, *Cordyceps* is still relatively considered safe and non-toxic for human consumption [22].

## 9. Conclusions and Future Perspectives

Over the past few years, people have shown confidence and trust in naturally occurring food-based products such as nutraceuticals for the treatment or management of various chronic diseases. Medicinal mushrooms have been proved to be very effective against numerous serious ailments. *Cordyceps*, some of the most important medicinal mushrooms, have been in use in the traditional Chinese medicine system since a long time ago. They are considered an excellent reservoir of various bioactive components, of which cordycepin is believed to have the most nutraceutical potential. Based on several research reports, cordycepin indicates its nutraceutical values by showing possible therapeutic activity against various diseases by modulating a number of cellular signaling pathways due to its redox behavior. In future, it would be important to uncover other unknown molecules present in *Cordyceps* and to understand their therapeutic potential. Likewise, it is equally important for the scientific community to explore the possibilities of nano-biotechnology-mediated targeted drug delivery systems for cordycepin and how to enhance its bioavailability. Further experimental and clinical studies are required to identify the exact mechanism behind the role of cordycepin, as well as its efficacy and safety alone and in combination. Since *Cordyceps* are edible, they will play a key role in the prevention and cure of various ailments caused by metabolic disorders or infections.

## Figures and Tables

**Table 1:** List of bioactive components present in *Cordyceps* with their biological activities and implication in therapeutics.

Bioactive Component	Biological Activity/Therapeutic Effect	References
***Nucleosides***		
Cordycepin	Antitumor, anti-diabetic, anti-inflammatory, antimicrobial, inhibit platelet aggregation, hypolipidemic, analgesic, immunomodulatory	[15,30,31,32]
Adenosine	Anticonvulsant, Anti-inflammatory, Anti-tumor	[15,29,33]
***Polysaccharides***		
Exopolysaccharide Fraction (EPSF)	Anti-tumor, antioxidant, anti-inflammatory, Immunomodulatory	[15,33,34]
Acid polysaccharides (APS)	Antioxidant, Immunomodulatory effect	[33]
CPS-1	Antioxidant	[15,33]
CPS-2	Cell proliferation inhibition	[33]
Mannoglucan	Cytotoxicity activity	[34]
CME-1	Antioxidant	[33]
PS-A	Inhibitory activity against cholesterol esterase	[34,35]
Cordyglucan	Anti-tumor	[33]
D-mannitol or Cordycepic acid	Diuretic, anti-tussive and anti-free radical activities	[2,29,31]
***Sterols***		
Ergosterol	Antimicrobial, antiviral, anti-arrhythmic effects, Helps in bone development	[2,30,31,36]
β-Sitosterol	Protect from breast, colon and prostate cancer	[34,36]
H1-A	Immunoregulation	[33]
***Proteins, Amino acids and Polypeptides***		
CSDNase	DNA hydrolysis, nucleolytic properties	[33]
CSP	Fibrinolytic activity	[33,34]
Cordymin	Anti-diabetic effect, antifungal	[22,33,37]
Cordycedipeptide A	Cytotoxic activity	[33,38]
Cordyceamides A and B	Cytotoxic activity	[34,38]
Tryptophan	Serves as precursor for the synthesis of the neurotransmitter’s serotonin and tryptamine	[33,39]
***Others***		
Xanthophylls	Anticancer	[30,31]
Fibrnolytic enzyme	Treatment of thrombosis	[30,31]
Proteoglucans	Enhanced anticancer effect on bladder cancer cells	[28,40]
Phenolic compounds	Antioxidant, antimicrobial, anti-arthritic, anti-carcinogenic, anti-hypertensive, cardio-protective, anti-inflammatory and anti-allergic	[28,41]
*N*-acetylgalactosamine	Necessary for intercellular communication	[22,42]
Exopolysaccharides	Nutraceutical, pharmaceutical	[22,43]
Chitinase, macrolides, cicadapeptins, myriocin, superoxide dismutase, protease, napthaquinone, cordyheptapeptide and dipicolinic acid	-	[22]
***Vitamins***		
B1	Essential in neurologic activities	[22,44]
B2	Helps in energy production	[22,44]
B12	Help in cellular metabolism, DNA synthesis, methylation and mitochondrial metabolism	[16,45,46]
E	Antioxidant, Help in formation of blood cells, muscles, lung and nerve tissues, Increase immunity	[16,44,45]
K	Essential for blood clotting	[22,44]

**Table 2:** Nutraceutical products from *Cordyceps* available on the global market.

Product Name	Health Benefit Claim	Manufacturer
Mycoformulas Endurance™	Enhancement of intracellular energy exchange, increases oxygenation and natural endurance	Myco Formulas, USA
Nutricafe-organic	Increases physical endurance and helps to remove toxins from your body	Aloha Medicinals USA
Mushroom Plus	Supporting immune system, energy levels and cognition	Link Nutrition Ltd., UK
Dragon Herbs	Increases the primary stimulating force for life activities	Iherb Holdings LLC, USA
OM™ Maitake	Supports weight control and blood sugar balancing	Yukiguni Maitake CO., LTD, Japan
Host Defense Mushrooms	Energy support	Host Defense, USA
CaféCeps^®^ Packets	Numbers of health-enhancing properties,	Madre Labs LLC, France
Bhutan *Cordyceps* Tea	Immunity booster, Anticancer, Anti-aging, Antioxidant, Improve kidney and gastrointestinal systems	Bhutan Natural, Singapore
MRM *Cordyceps* CS-4 Strain	It strengthens the immune system, respiratory system and cardiovascular system, Strengthens the natural metabolism of energy	All Star Health, USA
Ultra *Cordyceps* Plus	Help boost physical energy and stamina, improve vitality, support lung health, liver function, memory and mental ability	Doctor’s Best, USA
Now Foods *Cordyceps*	Immune health support	Now Foods, USA
Exploding Buds *Cordyceps Sinensis*	Immune health support	Iherb Holdings LLC, USA
Fungiology from California Gold Nutrition	For healthy immunity and health promotion of the entire body	California Gold Nutrition, USA
Planetary Herbals *Cordyceps* POWER CS-4	Energy support	Michael Tierra, USA
*Cordyceps* Capsules, Extracts or Powders	Energy support and stamina	Host Defense Mushrooms, USA
*Cordyceps* active	Promotes mental health, Ensure perfect oxygenation of the heart and vascular system	Terezia, Czech
Collagen C ReLift Capsules	For less wrinkles and improved complexion	Zein Pharma, Germany
MycoNutri *Cordyceps* Organic	Immune system support	The Really Healthy, UK
